# Microbial Fermentation Processes of Lactic Acid: Challenges, Solutions, and Future Prospects

**DOI:** 10.3390/foods12122311

**Published:** 2023-06-08

**Authors:** Yueying Huang, Yu Wang, Nan Shang, Pinglan Li

**Affiliations:** 1 Key Laboratory of Functional Dairy, College of Food Science and Nutritional Engineering, China Agricultural University, Beijing 100083, China; 2 College of Engineering, China Agricultural University, Beijing 100083, China

**Keywords:** lactic acid production, microbial fermentation, production costs, difficulties in production process

## Abstract

The demand for lactic acid and lactic acid-derived products in the food, pharmaceutical, and cosmetic industries is increasing year by year. In recent decades, the synthesis of lactic acid by microbials has gained much attention from scientists due to the superior optical purity of the product, its low production costs, and its higher production efficiency compared to chemical synthesis. Microbial fermentation involves the selection of feedstock, strains, and fermentation modes. Each step can potentially affect the yield and purity of the final product. Therefore, there are still many critical challenges in lactic acid production. The costs of feedstocks and energy; the inhibition of substrates and end-product; the sensitivity to the inhibitory compounds released during pretreatment; and the lower optical purity are the main obstacles hindering the fermentation of lactic acid. This review highlights the limitations and challenges of applying microbial fermentation in lactic acid production. In addition, corresponding solutions to these difficulties are summarized in order to provide some guidance for the industrial production of lactic acid.

## 1. Introduction

**Figure 1:**
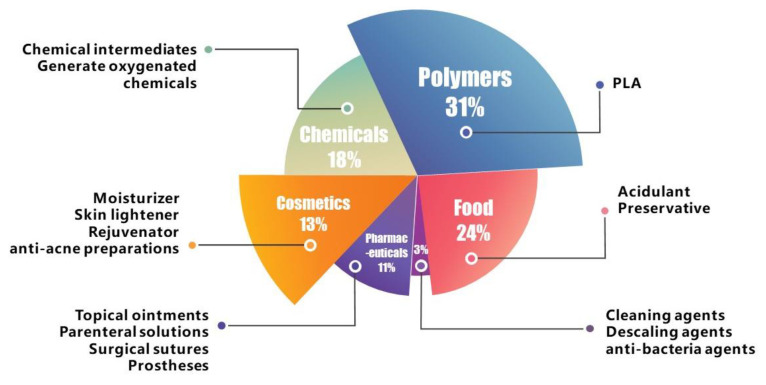
The applications of and demand for lactic acid in different industries.

Lactic acid (LA) is an essential organic acid with several applications in food, pharmaceuticals, and cosmetics [1,2]. Furthermore, LA is the monomer for synthesizing polylactic acid (PLA) plastic, a bioabsorbable and compostable plastic. PLA plastic has been considered one of the solutions which can be used to alleviate the “white pollution” caused by the massive use of petroleum-based plastics. It is an excellent substitute for conventional plastics in various applications, such as food packaging, field vegetable mulch, and surgical sutures. The widespread use of LA has led to a surge in its demand (Figure 1). The total demand for LA is expected to reach 19.601 million tons by 2025, which is estimated to be worth around USD 9.8 billion in the international market [1,3].

**Figure 2:**
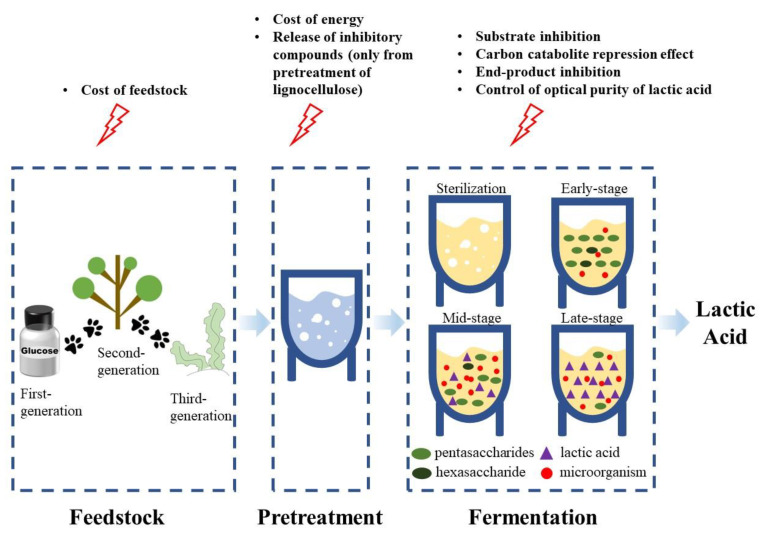
Current challenges in microbial production of lactic acid.

LA exists in two optical configurations: L-LA and D-LA. D-LA cannot be metabolized by humans and may even cause poisoning. DL-LA is a mixture of D-LA and L-LA. It is not only unavailable for food and pharmaceuticals, but also does not meet the requirements for the production of PLA plastics. Therefore, the pure isomer (L-LA or D-LA) is more valuable for utilization than the DL-LA [4,5]. LA can be synthesized by biological or chemical methods. The most common method of chemical synthesis is the hydrolysis of lactonitrile with strong acids, while biosynthesis uses microbial fermentation techniques. Compared with chemical synthesis, the synthesis of LA via microbial fermentation is gaining increasing attention from researchers due to advantages such as the low cost, high efficiency, environmental friendliness, and high optical purity of the product [6]. At present, the common microorganisms used to ferment LA mainly include *Lactobacillus*, *Bacillus*, *Escherichia coli*, *Rhizopus oryzae*, *Aspergillus niger*, *Saccharomyces cerevisiae*, *Lactobacillus cruzi*, etc. [7]. Although LA produced by microbial fermentation is superior to that produced through chemical synthesis, several challenges still remain (Figure 2). In terms of cost, the feedstocks initially used for LA fermentation were refined sugars or starches, which are more expensive. After that, to promote sustainability, agricultural by-products became the new substrate choices for LA fermentation. Agricultural by-products, such as straw, rice husk, bran, etc., are rich in cellulose and can be hydrolyzed into microbially available sugar to further produce high value-added products, such as LA. However, it was found that the production of inhibitory compounds after the pretreatment of these materials and the production of mixed sugars that the strains could not fully utilize caused a decrease in LA production [8,9]. There are also a number of hindering factors in the fermentation process, such as high initial sugar concentration, which inhibits the growth of the strain [10]. The accumulation of LA in the late stage of fermentation has a toxic effect on microbial cells [11]. By-products are produced and the optical purity of LA is reduced due to changes in the cultural environment and culture conditions [12]. Therefore, to meet the growing demand for LA, there is a requirement to overcome the hindrances to LA fermentation and develop lower-cost and more efficient technologies to produce LA with high optical purity. The main objective of this review is to discuss the current challenges faced by the microbial production of LA and to introduce possible solutions to overcome these challenges, with the aim of providing some guidance for the industrial production of LA.

## 2. Ways to Reduce the Costs of LA Fermentation

### 2.1. Inexpensive Feedstock Application for Fermentation

In LA production, feedstock costs usually account for 40–70% of the total costs [13]. To minimize the costs, carbon feedstock that can be fermented to yield LA has been investigated in various studies. The development of feedstock has progressed through three stages. The first generation was feedstock made with refined sugar or starch crops; second-generation feedstock consisted of lignocellulose biomass or other waste resources derived from food production and daily life; and third-generation feedstock comprises microalgae or macroalgae taken from the ocean, which is easily cultured in captivity. First-generation feedstocks are unsuitable for mass production of LA due to their competitive relationship with human food and their high costs [4]. The second-generation feedstocks are the most widely researched; these include by-products of agricultural products such as straw, bagasse, corn cobs; waste from food processing such as molasses and cheese whey; and food waste from life, such as expired bread and fruit peels. These substances can usually be hydrolyzed to produce large amounts of sugar for microbial fermentation into LA. For instance, sugarcane bagasse contains 35–40% cellulose and 25–30% hemicellulose, which can be hydrolyzed to hexoses and pentoses. *Bacillus coagulans* DSM ID 14-300 fermented acid-hydrolyzed bagasse at 52 °C to obtain LA at a concentration of 55.99 g/L with a productivity of 1.7 g/(L·h) and an optical purity of 99.4% [14]. In another study, corn cobs were used for LA production. The quantity of LA synthesized by *B. coagulans* H-1 was 0.85 g/g after 36 h of batch fermentation [15]. Molasses, the waste stream from the sugar process, is also a suitable substrate for LA fermentation. The cost of fermentation using molasses and corn pulp powder was estimated to be only 37.7% of the cost of production using an MRS medium. Microbial consortium CEE-DL15 utilized sugarcane molasses to produce LA. After 25 h of batch fermentation, 112.34 g/L LA was obtained from 350 g/L sugarcane molasses, with a maximum productivity of 4.49 g/(L·h) [16]. Whey, a waste product of the cheese production process, also contains about 49% lactose. Juodeikiene, et al. [17] used β-galactosidase for the enzymatic digestion of lactose in cheese whey. A maximum acid production of 47.0–51.2 g/L was achieved after whey fermentation by *Pediococcus acidilactici* KTU05-7 and *Pediococcus pentosaceus* KTU05-9 at pH 4.4–4.5 with the addition of 2% (*w*/*v*) neutralizer CaCO_3_. In addition, solid waste disposal has also been a key research issue in recent years. Food waste is rich in nutrients, including 30–60% starch, 5–10% protein and 10–40% lipids, and is a good substrate for LA production [18]. Yousuf et al. [19] obtained LA from food waste without an extra inoculum or pH control, with a yield of 0.46 g/g- total solids at 37 °C. Similarly, the highest output of 30.25 g/L LA was achieved from household food waste with *Lactobacillus rhamnosus* ATCC 7469 [20]. A variety of lignocellulose-based biomasses have been successfully applied in LA fermentation. However, these agricultural by-products are affected by a series of factors, including season, climate, and region. They may also produce some inhibitory substances during the pretreatment process, which may affect the industrial production of LA. The third-generation feedstock solves these problems to some extent. Microalgae and macroalgae have short growth cycles and are easy to cultivate. They are rich in carbohydrates and do not contain lignin, which is unavailable to microorganisms [21]. The overall quantity of LA synthesized from *Ulva fasciata*, *Gracilaria corticata,* and *Kappaphycus alvarezii* by *Lactobacillus plantarum* MTCC 1407 were, respectively, 0.4 g/g, 0.66 g/g, and 0.66 g/g DW of seaweed [22]. Nagarajan et al. [23] compared the ability of *Lactobacillus plantarum* to ferment glucose, sugar cane bagasse, cheese whey, and microalgae for LA production. Microalgal hydrolysate attained the highest productivity (12.56 g/(L·h) in continuous fermentation), along with great LA concentrations (37.9 g/L) and yields (0.91 g/g). However, in most studies on the fermentation of LA from algae, the yield is generally found to be lower than that of lignocellulosic biomass. How to increase the efficiency of LA production from algae will be the focus of future research. In general, a variety of inexpensive feedstocks are available for microbial fermentation to produce LA with high yields.

Table 1 summarizes the collection of LA concentrations, yields, and productivity levels by applying different lactic acid-producing bacterial strains and fermentation strategies using various feedstocks.

### 2.2. Change the Production Process to Save Energy Consumption

Traditional microbial fermentation generally requires sterilization and cooling of the medium, both of which consume a significant amount of energy and increase the cost of production. Therefore, researchers have attempted non-sterilized fermentation for LA production. Since most LA producers are used at medium temperatures, non-sterilization fermentation can easily lead to contamination. Anaerobic fermentation is one of the ways to avoid the growth of miscellaneous bacteria. For example, a high D-LA concentration (113.18 g/L) with an average productivity of 2.36 g/(L·h) was obtained in fed-batch fermentation from cheese whey (without sterilization) at 42 °C in an anaerobic environment [32]. In addition, the simultaneous enzymatic saccharification and fermentation of biomass can reduce energy costs. With simultaneous saccharification and fermentation (SSF), not only can energy costs be reduced by avoiding the significantly energy-consuming biomass saccharification, but also by providing sufficient sugar release to achieve higher productivity than single-step conversion. However, SSF is less efficient due to the low activity of saccharifying enzymes at mesophilic temperatures. Therefore, microorganisms that can ferment at thermophilic temperatures are widely used in this fermentation process. They can function at higher temperatures than those used in normal microbial fermentation, and have a good ability to produce LA. One study reported that a strain of *Enterococcus faecium* QU 50 isolated from Egyptian fertile soil was effective in fermenting xylose to produce LA in wide pH (6.0–10.0) and temperature (30–52 °C) ranges. In this study, the maximum yield of LA (1 g/g) with an optical purity ≥99.2% was attained at 50 °C [36]. Similarly, *B. coagulans* LA204 achieved an LA yield of 0.54 g/(g corn cob), with an average output of 0.57 g/(L·h), using corncob and corn steep liquor powder as substrates with fed-batch simultaneous saccharification and fermentation [37]. Another study conducted 5 L and 50 L bioreactor scale-up trials and found that the SSF of corn stover by the *P. acidilactici* DQ2 at 48 °C still achieved a high LA concentration (~101 g/L) and high productivity (~1.06 g/(L-h)), demonstrating its strong potential for industrial applications [38]. Furthermore, a recent study achieved simultaneous liquefaction, saccharification, and fermentation. *Enterococcus faecalis* DUT1805 had superior thermotolerance and was active at higher temperatures (50 °C or higher). In addition, the highest output—73.75 g/L LA with a yield of 87% of initial starch—was attained by aging paddy rice with hull [39]. If this process can be successfully applied in industry, it will save much time and energy during production. Moreover, searching for strains that can produce LA directly from starchy material can also save energy consumed during the saccharification step [40]. Overall, the researchers intend to minimize energy costs by simplifying and optimizing the fermentation process.

## 3. Problems in the Fermentation Process and Their Solutions

### 3.1. Select Appropriate Strains and Fermentation Processes to Accommodate High Concentrations of Substrates

Traditional split-wholesale fermentation is performed by optimizing the substrate in the medium to the optimum concentration of the strain so that it ferments as much product as possible in the experimental environment, but this is often time-consuming and energy-intensive. In order to increase the production efficiency, it is desired that the strains produce large amounts of LA in a medium with high substrate concentration. However, high substrate concentrations may suppress the strain, as evidenced by extended lag periods, increased osmotic stress, etc. [10]. Therefore, several studies have addressed the inhibition of strains at high substrate concentrations by screening and mutating the strains, as well as changing the fermentation process. The mutant strain *Lactobacillus paracasei* NCBIO01-M2 was obtained by atmospheric pressure room-temperature plasma treatment and high-throughput screening technology. It can flexibly regulate the ratio of unsaturated fatty acids and intracellular compatible solute pools, and, therefore, has a high tolerance to osmotic stress. The 248 g/L glucose in the medium was wholly consumed during the batch fermentation, producing 223.7 g/L LA with a yield of 5.53 g/(L·h) [41]. As for the production process, fed-batch fermentation and SSF are two processes with mature applications at present. Fed-batch fermentation leads to a higher utilization of the substrate compared to continuous fermentation. It provides a suitable growth environment for the bacteria by controlling the concentration of nutrients during fermentation so that the production status of the bacteria can be maintained at optimal levels. Pejin et al. [24] reported the production of L-LA by *L. rhamnosus* ATCC 7469 from brewer’s spent grain hydrolysate and malt rootlet extract. The highest LA concentration in the batch fermentation was 25.73 g/L, with a yield of 0.95 g/(L·h) and a conversion of 86.31%; on the other hand, an increase in LA concentration to 58.01 g/L, an increase in yield to 1.19 g/(L·h), and a conversion rate of 88.54% was achieved with fed-batch fermentation. SSF enabled the timely utilization of sugars from starch hydrolysis, and the hydrolysis reaction proceeded positively. Using this fermentation mode, the initial sugar concentration in the medium was low. Additionally, the degree of hydrolysis of polysaccharides was increased, which facilitated the growth and fermentation of microorganisms. The shortcoming of this process is that saccharification requires a high temperature. Therefore, high-temperature-tolerant strains need to be selected to meet this process. The strain *B. coagulans* WCP10-4, isolated from soil, could be fermented by SSF at 50 °C, consuming 200 g/L of corn starch to produce 202.0 g/L of L-LA [42]. In conclusion, strains with high glucose tolerance, as well as SSF and fed-batch fermentation, have great advantages in terms of LA production.

### 3.2. Methods to Control the Carbon Catabolite Repression Effect

The substances obtained by hydrolysis of raw materials, such as lignocellulose, are usually a mixture of hexoses and pentoses. Most strains have a carbon catabolite repression (CCR) effect on the fermentation of mixed sugars, which affects the utilization of sugars and reduces the final production of LA [43]. CCR can be mitigated either by the selection of production strains or by adjusting the composition of the medium. First, CCR-negative strains can be selected as inoculants. The metabolism of *Lactobacillus lactis* 2369 (CCR negative) was shown to follow a mixed sugar fermentation pathway during the fermentation of rice straw. Glucose was its preferred substrate, fully utilized within 48 h, followed by xylose utilization of more than 40% at the end of fermentation [44]. Secondly, mixed bacterial fermentation using microbial consortia can exploit the abilities of different strains to consume different sugars and alleviate the CCR effect. The microbial consortia isolated from cattle stomach were domesticated to obtain the microbial consortia DUT47, which has robust tolerance to inhibitors. In the experiment, the microbial consortia DUT47 simultaneously utilized glucose and xylose. LA was produced from corn stover under non-detoxified, non-sterile conditions, with a yield of 0.50 g/(g corn stover) and a productivity of 0.32 g/(L·h) [45]. In general, most studies have focused on rescuing the CCR by screening or modifying strains to adapt them to the composition of the medium. Recently, several studies have proposed another feasible option to eliminate the CCR of microorganisms by adjusting the composition and the ratio of the substrate. *E. faecium* QU 50 exhibited a significant CCR in the fermentation of glucose/xylose mixtures, with a much lower xylose consumption ratio than glucose. However, CCR was avoided in the fermentation of cellobiose/xylose mixtures. *E. faecium* QU 50 can simultaneously utilize various sugars in a mixed sugar medium consisting of 5 g/L glucose, 40 g/L cellobiose, and 20 g/L xylose. In high-density open continuous fermentation with cell cycling technology, the productivity of LA was 6.49 g/(L·h). No by-products were detected up to 250 h of fermentation, and the strain was stable for up to 500 h of fermentation [46]. The researchers reached a slightly different conclusion in another study than that mentioned above. The study tested the ability of the strain to ferment cellobiose/glucose mixtures. The results showed that cellobiose promoted the consumption of glucose, but glucose inhibited the consumption of cellobiose, and there was a significant CCR. Therefore, a medium with glucose and xylose as the main sugars along with a small amount of cellobiose is suitable for LA fermentation of *B. coagulans* Azu-10 [47]. In short, to effectively mitigate the CCR, the formulation of the medium can be designed in a targeted manner according to the sugar utilization characteristics of the producing strains.

### 3.3. Release of Inhibitory Compounds during Feedstock Pretreatment and the Detoxification Methods

The pretreatment of lignocellulose generates some inhibitory compounds, including furan derivatives, weak acids, and phenolic compounds [48]. Furan derivatives include 5-hydroxymethylfurfural (5-HMF) and furfural, which negatively impact cell growth and sugar uptake [49]. Phenolic compounds such as vanillin, syringaldehyde, and ferulic and coumaric acid have been shown to have inhibitory effects on cellulolytic enzymes, DNA transcription, and protein synthesis [1,50]. Weak acids such as acetic and formic acid can hinder the growth of microbial cells [51]. The traditional way to remove these compounds is to wash them with several times their volume in water, but this is not easily implemented in industry. In recent years, various physical, chemical, and biological methods have been applied to remove inhibitory substances, showing promising results [37,52,53]. Although the removal of inhibitory substances from the medium allows the production strain to ferment properly, this step also increases the cost of production. The detoxification step can be omitted by developing strains capable of tolerating a wide range of concentrations of the inhibitory compounds. Adaptive evolution of the strain is one way to enhance its tolerance. *B. coagulans* was inoculated with a 20% volume fraction of wheat straw hydrolysate for a passaging culture. After 63 generations, the strain was stable in 45% (*v*/*v*) hydrolysate, while the parental strain failed to produce LA at concentrations above 25% (*v*/*v*) hydrolysate [54]. Similar domestication was equally effective for the microbial consortia [45]. In addition, finding production strains that are both able to produce LA and degrade inhibitors can also allow their effect on LA production to be avoided. The first use of *B. coagulans* for LA production and bio-detoxification was tested by Alves et al. [14]. The LA yield of sugarcane bagasse hemicellulose hydrolysate, fermented by *B. coagulans* DSM ID 14-300, was 0.87 g/g, while the removal of 5-hydroxymethylfurfural and furfural from the hydrolysate reached 99.6% and 100%, respectively. Moreover, the anaerobic environment may also enhance the tolerance of the strain to inhibitory compounds [55].

### 3.4. Suitable Strains and Fermentation Processes for Solving Product Inhibition Problems

The accumulation of the end product, lactic acid, during fermentation affects the metabolism of microorganisms, and cytotoxicity occurs around the pKa value (3.86) [56]. Reducing the effect of end-product inhibition can be achieved by increasing the acid tolerance of the strain and optimizing the fermentation process. The end-product LA causes the pH of the medium to drop significantly. Therefore, the industry maintains the pH at 5.5–6.5 by adding neutralizers such as Ca(OH)_2_ and CaCO_3_. However, this increases the difficulty and cost of subsequent LA isolation and purification [57]. Improving the acid tolerance of strains can be achieved by adaptive evolution or genetic modification. Adaptive evolution of *S. cerevisiae* SR8LDH was carried out in a medium (pH 3.1) containing 20 g/L glucose and 8% LA. The evolved strain *S. cerevisiae* BK01 was fermented at a high sugar concentration (200 g/L), and the LA yield was increased by 17% [58]. After the adaptive domestication of *P. acidilactici*, the amount of bacteria doubled when the pH of the fermentation broth was reduced to 4.0 [59]. Gene knockdown by RNA interference allowed for sequence-specific down-regulation on a genome-wide basis, according to the phenotype. It achieved slight down-regulation of multi-copy genes without considering the genome’s copy number. The identified acid-tolerant gene target was transferred to the lactic acid-overproducing strain *S. cerevisiae* CEN.PK in the form of a knockout, resulting in a 33% increase in cell growth, a 58% increase in glucose consumption, and a 60% increase in L-LA production [60]. Strains tolerant to acidic environments due to their structures are also promising for LA production. *S. cerevisiae* is tolerant to acid. The D-LA-producing strain *S. cerevisiae* JHY5210 was obtained by expressing the D-lactate dehydrogenase gene (*Lm. ldhA*). Genes associated with ethanol production (*ADH1* and *PDC1*), glycerol production (*GPD1* and *GPD2*), and D-lactate degradation (*JEN1* and *DLD1*) were also deleted from the strains to reduce by-product production [61]. However, 0.6 g/g of ethanol was still produced during the fermentation of *S. cerevisiae* JHY5210. In the following study, *S. cerevisiae* JHY605, which was missing five *ADH* genes, *GPD1,* and *GPD2*, was used as the starting strain. To further improve the traits of the strain, *DLD1* was deleted and *TDH3* was integrated into its locus as a promoter to control *Lm. ldhA*. The final strain, JHY5610, was obtained with a 44% higher level of D-LA production. By adaptive evolution of the JHY5610 strain, the D-LA yield of the obtained strain, JHY5710, was again enhanced by 30% [62]. In summary, the combination of adaptive evolution and genetic modification can substantially increase the acid tolerance of the strain, resulting in efficient conversion of the carbon source to LA.

In terms of process, fermentation processes such as continuous fermentation, fed-batch fermentation, cell immobilization fermentation, and cell-recycled batch fermentation have been developed and applied to LA fermentation. These strategies reduce the effect of end-product accumulation in the fermentation system on the strain. Continuous fermentation is the continuous addition of fresh medium to the bioreactor at a specific rate while allowing the fermentation broth containing metabolites to flow out so that the yield of the fermentation system is maintained at a high level. The maximum LA productivity of 3.69 g/(L·h) was reached at a pH of 6.0, a temperature of 50 °C, and a dilution rate of 0.167 h^−1^ [63]. However, continuous fermentation may also have the disadvantage of inadequate utilization of nutrients. Fed-batch fermentation leads to a higher utilization of the substrate compared to continuous fermentation. It provides a suitable growth environment for the bacteria by controlling the concentration of nutrients during fermentation so that the production status of the bacteria can be maintained at optimal levels. Pejin et al. [24] reported the production of L-LA by *L. rhamnosus* ATCC 7469 from brewer’s spent grain hydrolysate and malt rootlet extract. The highest LA concentration in the batch fermentation was 25.73 g/L, with a yield of 0.95 g/(L·h) and a conversion of 86.31%, while an LA concentration increased to 58.01 g/L, a yield increased to 1.19 g/(L·h), and a conversion of 88.54% were achieved with fed-batch fermentation. Cell immobilization technology was utilized to immobilize microbial cells on water-insoluble carriers, allowing them to carry out their life activities in a specific spatial range. It has the advantage of being less susceptible to contamination, and has a high fermentation rate. It has been shown that cell immobilization technology in batch fermentation and continuous fermentation results in relatively high LA yields [64]. However, it has also been pointed out that cell immobilization bioreactors may decrease productivity after long-term use due to limited mass transfer and accumulation of dead cells [65]. Furthermore, the cell-recycled batch fermentation process is also a way to reduce product inhibition. After a period of fermentation, the product is promptly removed, and a new batch of fermentation broth is added to reuse the bacteriophage cells. This fermentation mode omits the inoculant preparation step and eliminates the lag period of cells, thus reducing the fermentation time. Using membrane-integrated repeated batch fermentation, *B. coagulans* IPE22 was fermented with wheat straw hydrolysate as the carbon source. The LA productivity increased from 1.01 g/(L·h) to 2.35 g/(L·h) after six fermentation batches. It was speculated that the synergistic effect of cell reuse and microbial domestication may have improved the quality of the strain and facilitated its fermentation of the sugar mixture [66]. Furthermore, the insertion of dynamic membranes into the fermentation reactor allowed the microbial cells to be retained and reused, and the permeability of the dynamic membranes can be maintained by regular backwashing [67]. Because of the high cost of processing equipment for cell immobilization technology and cell cycling technology, the application of these method is still in the laboratory stage.

### 3.5. Methods to Control of Optical Purity of Lactic Acid

The stereospecificity and optical purity of LA produced by microbial fermentation depend mainly on the chosen strain and its lactate dehydrogenase (LDH) specificity. It has been found that both L-LDH and D-LDH enzymes can be active in some LA fermentation strains, producing LA with low optical purity [68]. Improving the optical purity of LA in fermentation products can be achieved by genetic modifications and fermentation conditions of the strain. Temperature is one of the key factors affecting the fermentation of strains for the production of L-LA and D-LA. One study tested the optical purity of LA produced by fermenting carbohydrates using activated sludge (a by-product of municipal wastewater treatment plants) as an inoculant at different temperatures. It was found that both the production and consumption of L-LA and D-LA are optimal at moderate temperatures, while L-LA consumption and D-LA production are suppressed at high temperatures. Therefore, the authors developed a two-stage, temperature-controlled method, i.e., high-temperature preheating at 50 °C for 4 h, followed by medium-temperature fermentation at 37 °C for 34 h, resulting in L-LA with an optical purity of 98.3% [69]. Another study also showed that temperature affects the strains’ selectivity in the production of L-LA and D-LA. *Lactobacillus coryniformis*, which is high in D-LA production, shows a sharp increase in L-LA concentration at high temperatures [70]. Modifying the strain genes can directly control the production of either pure L-LA or pure D-LA. Kuo et al. [12] blocked the metabolic pathway of D-LA by knocking out the D-lactate dehydrogenase from *L. paracasei*. The obtained *ldhD*-deficient strain 7BL was able to produce optically pure L-LA, and no D-LA production was detected during its fermentation of lignocellulosic hydrolysate.

## 4. Selecting Suitable Fermentation Processes for LA Production

The conventional two-step batch fermentation method has the disadvantages of substrate and/or product inhibition and high energy consumption. In order to solve these problems and improve production efficiency, some fermentation processes have been developed and applied in practical production (these are described in Section 3.1 and Section 3.4). This includes fed-batch fermentation, continuous fermentation, cell immobilization fermentation, and cell-recycled batch fermentation. These can be used to address some of the challenges encountered in LA production. Since each of these processes has advantages and disadvantages, they are applicable in different situations. All substrates are converted into a fermentable form before fermentation, so the type of substrate has little influence on the choice of fermentation process. It is worth noting that inhibitory compounds released during the pretreatment of lignocellulose may not be completely removed. Therefore, this may affect the strain growth, and cell immobilization fermentation and cell-recycled batch fermentation are not recommended. According to the characteristics of these processes, the different ranges of applications are listed in Table 2.

## 5. Conclusions

The main challenge of LA production is achieving high LA concentrations, yields, productivity, and optical purity using economical renewable resources. The cost of LA production can be significantly reduced by selecting suitable raw materials and fermentation strategies. While facing a fermentation process that is hindered by substrate concentration, product concentration, and the presence of inhibitory compounds, strains must be screened for better tolerance or domesticated to adapt to the fermentation environment. In addition, the continuous development of new processes and the creation of a suitable production environment for the production strains are also critical to guarantee LA yields and production rates. Therefore, exploring excellent production strains and designing efficient processes will be the focus of future research on microbial fermentation for LA production.

## Figures and Tables

**Table 1:** Recent studies on lactic acid production from low-cost carbon sources.

Feedstock	Strain (s)	Strategy	Lactic Acid			References
			Concentrations (g·L)	Yield (g·g^−1^)	Productivity (g·(L·h) ^−1^)	
Brewer’s spent grain, malt rootlets	*L. rhamnosus* ATCC 7469	Batch fermentation	25.73	-	0.95	[24]
		Fed-batch fermentation	58.01	-	1.19	
Beechwood	*Lactobacillus delbrueckii* subsp. *bulgaricus* (ATCC^®^ 11842)	Simultaneous saccharification and fermentation (batch)	62.00	0.69	0.86	[25]
Food waste	*E.coli* JU15	Batch fermentation	22.66	0.99	0.73	[26]
Food waste	*Lacobacillus manihotivorans* DSM 13343	Batch fermentation	18.69	0.73	-	[27]
	*L. plantarum* DSM 20174		17.03	0.69	-	
Orange peel waste	*Lactobacillus delbrueckii* ssp. *bulgaricus* CECT 5037	Batch fermentation	39.00	-	-	[28]
Coffee ground waste	*L. rhamnosus* ATCC 10863	Batch fermentation	24.95	-	0.59	[29]
Sugarcane bagasse	*Lactobacillus pentosus* ATCC 8041	Batch fermentation	65.00	0.93	1.01	[30]
Sugarcane molasses	microbial consortium CEE-DL15	Batch fermentation	112.34	0.81	4.94	[16]
Bakery waste hydrolysates and lucerne green juice	*B. coagulans*	Batch fermentation	62.20	0.57	2.59	[31]
		Continuous fermentation	55.00	0.48	11.28	
Cheese whey powder	*Lactobacillus bulgaricus* CGMCC 1.6970	Batch fermentation	70.70	-	1.47	[32]
		Continuous fermentation	113.18	-	2.36	
*Sargassum cristaefolium*	*L. plantarum* 23	Batch fermentation	26.00	0.81	7.53	[33]
*Ulva sp.*	*L. rhamnosus*	Batch fermentation	30.93	0.85	7.53	[33]
*Gracilaria* sp.	*Weissella paramesenteroides* 24	Batch fermentation	29.00	0.94	3.58	[33]
*Eucheuma* *denticulatum*	*Bacillus coagulans* ATCC 7050	Prehydrolysis simultaneous saccharification and fermentation (batch)	14.00	-	-	[34]
*Hydrodictyon reticulum*	*Lactobacillus paracasei* LA104	Simultaneous saccharification and cofermentation	37.11	0.46	1.03	[35]

**Table 2:** Fermentation processes for various pretreated substrates and their scopes of application.

Fermentation Process	Feedstock	Pretreatment	Applicable Scope
Fed-batch fermentation	First-generation feedstocks	No need	▪ High concentrations of substrates ▪ High acid tolerance of the strain
	Second-generation feedstocks	Solids (e.g., food waste, corn stover): mill (except powder)/dissolve/mix (with other base feedstocks)/detoxification (lignocellulose as substrate)/sterilization/saccharification (or SSF) Liquid (e.g., whey, molasses): mix (with other base materials)/sterilization/saccharification (or SSF)	
	Third-generation feedstocks	Mill/dissolve/mix (with other base materials)/detoxification/sterilization/saccharification (or SSF)	
Continuous fermentation	First-generation feedstocks	Same as above	▪ High-cell-density fermentation ▪ Low concentrations of substrates
	Second-generation feedstocks		
	Third-generation feedstocks		
Cell immobilization fermentation	First-generation feedstocks	Same as above	▪ Suitable for batch fermentation or continuous fermentation ▪ Fermentation environment and fermentor are strictly sterilized ▪ Pursuit of minimal fermentation time
	Second-generation feedstocks	Same as above (pretreatment of feedstocks (e.g., lignocellulose) that may produce inhibitory substances is not recommended)	
	Third-generation feedstocks	Same as above	
Cell-recycled batch fermentation	First-generation feedstocks	Same as above	▪ High-cell-density fermentation ▪ Fermentation environment and fermentor are strictly sterilized ▪ Pursuit of minimal fermentation time
	Second-generation feedstocks	Same as above (pretreatment of feedstocks (e.g., lignocellulose) that may produce inhibitory substances is not recommended)	
	Third-generation feedstocks	Same as above	

## Data Availability

Data are contained within the article.
